# The Dietary Inclusion of Corn and Peas Affects the Fecal Bacterial Diversity and Composition of Sarda Goats

**DOI:** 10.3390/ani16162486

**Published:** 2026-08-10

**Authors:** Tiziana Maria Mahayri, Giuseppe Moniello, Jakub Mrázek, Simona Kvasnová, Giuseppino Cocco, Giovanni Garippa, Kateřina Olša Fliegerová

**Affiliations:** 1 Laboratory of Anaerobic Microbiology, Institute of Animal Physiology and Genetics, Czech Academy of Science, 14220 Prague, Czech Republic; mahayri@iapg.cas.cz (T.M.M.); mrazek@iapg.cas.cz (J.M.); simona.zerebna@natur.cuni.cz (S.K.); fliegerova@iapg.cas.cz (K.O.F.); 2 Department of Veterinary Medicine, University of Sassari, 07100 Sassari, Italy; giovannigarippa@gmail.com; 3 Faculty of Science, Charles University, Albertov 6, 12800 Prague, Czech Republic; 4 Independent Researcher, 09071 Abbasanta, Italy; g.cocco0852@gmail.com

**Keywords:** feces, bacterial community, bacterial diversity, diet, goats

## Abstract

This study investigated the effects of dietary inclusion of corn and peas on the fecal microbiota composition and functional potential in Sarda goats. Twelve female goats were allocated to two groups, a group grazing on a natural pasture (GP) and a group grazing on the same pasture with a diet enriched with corn and peas (GC), both grain supplements at 100 g/day. Fecal samples were collected, freeze-dried and subjected to 16S rRNA gene sequencing of the V4–V5 region, followed by bioinformatic and statistical analyses. Alpha diversity metrics indicated a significant reduction in microbial richness and phylogenetic diversity in the GC group, and beta diversity analysis revealed distinct community clustering. Taxonomic profiling showed Firmicutes and Bacteroidota as the dominant phyla, with a significantly altered Firmicutes/Bacteroidota (F/B) ratio in the GC group. LEfSe analysis identified 31 differentially abundant taxa between the groups. Functional profiling revealed 28 KEGG Level 3 pathways enriched in both groups. Pathways related to cell growth and motility were more abundant in the GP group, while the GC was enriched in amino acid metabolism and membrane-associated processes. These findings suggest that dietary supplementation with plant-based concentrates modulates both the taxonomic structure and functional potential of the fecal microbiota in goats.

## Simple Summary

Feeding goats a small daily supplement of corn and peas changed the composition and activity of their gut microbiota. Goats that only grazed on pasture had richer and more diverse microbial communities, while goats receiving the grain mix showed a clear shift in which bacteria were present. The balance between major bacterial groups (Firmicutes and Bacteroidota) changed, and several specific taxa differed between the two diets. Functional analysis showed that pasture-fed goats had more pathways linked to cell growth and movement, whereas grain-supplemented goats showed more activity related to amino acid metabolism and membrane functions. Overall, adding plant-based concentrates to the diet noticeably altered both the structure and the metabolic potential of the fecal microbiota in Sarda goats.

## 1. Introduction

Ruminants play a crucial role in the global food sector as they produce significant amounts of high-quality animal protein in the form of milk and meat and have the unique ability to digest fibrous feedstuffs that are indigestible to humans. The ability of ruminants to convert low-quality plant material into nutrient-rich food makes them indispensable for the sustainability of agriculture [1,2]. Goats occupy an important position in livestock production, providing economic benefits through the production of meat, milk, leather and pelts [3,4,5] under harsher environmental conditions than other domesticated ruminants [6]. In particular, the ability to thrive well in arid areas is the reason for their wide distribution throughout the world, with an estimated population of over one billion goats in about 1000 recognized breeds worldwide [7]. In Italy, goat farming is a key agricultural sector, especially in Sardinia, where around 300,000 goats are kept, making an important contribution to the regional livestock industry. The Sarda goat is an autochthonous breed of the island that is well adapted to the semi-arid climate. Despite its economic importance, milk production in traditional Sarda goat farming remains relatively insufficient because animals are generally reared under extensive or semi-extensive grazing systems, allowing the goats to graze freely on Mediterranean scrubland. These systems are characterized by seasonal fluctuations in pasture quantity and quality [8,9,10]. Consequently, strategic dietary supplementation is frequently adopted to compensate for nutritional deficiencies and improve productive performance. These modifications include feeding concentrates to accelerate milk production and body weight gain [11]. However, such dietary strategies may have implications for the health and stability of the rumen, a critical organ in ruminants where microbial fermentation of feed takes place. The rumen harbors a dense and diverse microbial ecosystem that plays a central role in digestion, nutrient absorption, and immune function. It is also the primary site where diet-induced microbial shifts are first triggered [12]. High-grain (HG) diets, often used to increase productivity, can induce changes in the rumen microbial community [13,14,15] and may be associated with metabolic disorders [16,17], such as subacute ruminal acidosis (SARA) [18]. Disturbances in microbial populations are of particular concern as they affect not only animal health but also productivity and the quality of dairy products and meat [19]. It should be noted that concentrate supplementation is not naturally harmful as moderate levels are commonly used in extensive and semi-extensive goat production. This differs substantially from excessive grain feeding capable of inducing SARA. Therefore, the percentage of concentrate feed in the diet must be carefully selected [20].

The microbial community in the gastrointestinal tract (GIT) of goats, primarily composed of bacteria, has many important functional roles and significant effects on overall food digestion and nutrient absorption [4]. Although microbial changes begin in the rumen, these shifts can spread along the GIT and affect microbial communities further downstream, including those in the large intestine and feces [21].

Fecal sampling provides a non-invasive, simple, and cost-effective alternative for monitoring microbial changes induced by dietary interventions [22]. Although fecal microbial communities do not perfectly reflect those of the rumen [23,24,25], they are still significantly influenced by ruminal processes and dietary factors. Analysis of feces offers valuable insights into gut microbial shifts [26,27,28] and has been used as a proxy for digesta samples from the large intestine [29,30,31], as the hindgut microbiome plays an essential role in fiber fermentation and immune modulation in ruminants [32].

Literature data indicate that excessive grain feeding alters the structure of the fecal microbial community [28,31,33]. The results are consistent in that diversity is reduced, but the effects on microbiome composition vary depending on the species of animal and the amount and type of concentrate feed. In general, the increase in starch availability enhances populations of amylolytic bacteria, but since many different microorganisms possess amylolytic activity, it is difficult to predict which specific taxa will be most affected [34,35,36]. Moreover, the changes in fecal bacterial composition are very different when a moderate grain diet is offered without inducing SARA [28]. Concentrate feed is commonly used for ruminants as a source of energy and protein as well as a supplier of essential specific nutrients such as amino acids, fatty acids, enzymes, vitamins, and minerals. To optimize these beneficial impacts, it is crucial to study the effects of grain inclusion in forage-based diets on the microbiota composition under conditions that do not trigger SARA.

Firmicutes and Bacteroidetes are the dominant phyla in the feces of goats [37,38], but the bacterial composition at lower taxonomic levels depends on diet and other factors, such as breed, husbandry conditions, location, and health status. In the feces of grazing Sarda goats, the most abundant genera were *Ruminococcus*, *Eubacterium*, *Roseburia*, and *Clostridium* [39]. However, available information mainly describes the baseline microbial composition of this locally adapted breed, while the response of Sarda goat intestinal microbiota to dietary modulation remains largely unexplored. In general, grain-rich diets reduce bacterial diversity and richness [40]; however, the specific bacterial shifts related to the dietary changes vary considerably, depending on concentrate inclusion level, starch source, feeding duration, and animal breed. For example, different studies have reported a decline in the relative abundance of key fiber-degrading bacteria, whereas the abundance of *Prevotella*, *Rikenellaceae RG9 gut cluster*, *Christensenellaceae R-7*, *Butyrivibrio*, *Succiniclasticum* and *Lactobacillus* spp. has been shown to increase [41,42,43]. These findings are based on rumen microbiome analyses of goats fed relatively high doses of concentrate feed. The effect of appropriate doses of grain feed on the bacterial composition of feces is largely unknown. This represents an important gap because moderate concentrate inclusion is widely used to support energy and protein requirements, yet its microbial consequences remain poorly characterized. Furthermore, fecal microbiota deserves specific investigation since it is strongly shaped by digestive processes and provides a non-invasive, field-feasible way for monitoring microbial shifts in grazing animals. Investigating the response of the fecal microbiota of the locally adapted Sarda goat to moderate dietary supplementation provides a unique opportunity to explore the interaction between breed-specific microbial characteristics and nutritional modulation under practical grazing conditions.

Therefore, the present study aims to investigate the impact of dietary inclusion of corn and peas (100 g per day each) on the bacterial diversity, structure, and functional potential in the feces of Sarda goats. Unlike previous studies mainly focused on high-grain feeding and ruminal dysbiosis associated with SARA, the present work evaluates a low-dose supplementation strategy representative of semi-extensive Mediterranean farming conditions. The fecal bacteriome was analyzed by high-throughput sequencing (HTS) of 16S rRNA gene fragments and assessed for its taxonomic composition, diversity, and functionality. This study will contribute to a better understanding of how dietary modifications influence microbial communities and may provide insights into optimizing ruminant nutrition for improved productivity and health.

## 2. Materials and Methods

### 2.1. Animals and Sample Collection

This study was conducted on a private farm located in Sardinia (Italy, 39.0089° N, 8.8155° E), with the permission of the farm owners. Fecal samples were collected from 12 healthy female goats (aged 1–3 years) of the Sarda breed (body weight: 35 kg ± 1.71; BCS: 2.5 ± 0.16) to evaluate the effects of dietary supplementation on microbiota. Six samples obtained from goats grazing on natural meadow with shrubs of Mediterranean maquis (group GP, n = 6) were compared with six samples of goats on the same pasture but supplemented with 100 g of pea seeds and 100 g of corn per day (group GC, n = 6). Both groups grazed the same pasture under identical grazing management throughout the study, so that pasture botanical and nutritional variability affected the two groups equally. Corn and peas were provided individually to each goat in controlled amounts. The animals underwent a 30-day adaptation period, and the supplementation period lasted 45 days before fecal sample collection. The botanical composition of the pasture included a diverse range of plants; a dominant species was *Festuca* spp., followed by *Dactylis glomerata*, *Cistus* spp., and other characteristic Mediterranean forage species. Although the botanical composition of the pasture was documented, its chemical composition was not analyzed. Consequently, differences in forage nutrient composition could not be considered when interpreting the observed changes in the fecal microbiota. The nutritional composition of hay, corn, and pea seeds is presented in Table S1. The goats were maintained under semi-extensive conditions, with unrestricted access to natural pasture during the day from 7:30 to 9:30 a.m. and from 4:30 to 6:30 p.m. in relation to the period of the year and depending on seasonal forage availability. Water was freely available both outdoors and indoors. The animals were kept in a pen during the night, where a hay meadow was freely available for the animals, and its daily average intake was 300 g/head. The main characteristics of the animals, production system, and feeding regimen are presented in Table S2. The fresh fecal samples were collected immediately after defecation during the morning grazing of animals. Only the inner portion of each fecal pellet was sampled using sterile disposable gloves, in an appropriate way, avoiding bedding contamination by any material that had contacted soil, vegetation, or other environmental surfaces. The samples were put in a sterile bag and carried in a small portable refrigerator (8 °C) to the Department of Veterinary Medicine, University of Sassari (Sassari, Italy). Samples were immediately frozen (−20 °C), and after 2 days, a representative aliquot of each sample was freeze-dried using the Heto PowerDry LL3000 freeze dryer (Thermo Fisher Scientific, Wilmington, DE, USA). Moisture and dry-matter levels were quantified by recording the weight of each fecal sample before and after lyophilization. The dried samples were transferred to the Institute of Animal Physiology and Genetics of the Czech Academy of Sciences (Prague, Czech Republic) for subsequent analyses.

### 2.2. DNA Isolation

The fecal samples were disintegrated to homogeneity in liquid nitrogen using a mortar and pestle. A quantity corresponding to 300 mg of wet weight of sample was used for DNA extraction. The DNA was extracted using the DNeasy^®^ Plant Pro Kit (Qiagen, Hilden, Germany), then the concentration and quality of nucleic acids (ratio 260/280) were assessed using a NanoDrop 2000c UV-Vis spectrophotometer (Thermo Scientific, Wilmington, DE, USA), and the isolated DNAs were kept at −20 °C until further analysis.

### 2.3. PCR Amplification and Purification

The extracted DNAs were diluted 10 times with nuclease-free water, and 2 μL of the diluted DNA was utilized as a template for amplification. The V4–V5 hypervariable region of the bacterial 16S rRNA gene was targeted using the primers BactBF (GGATTAGATACCCTGGTAGT) and BactBR (CACGACACGAGCTGACG) as previously described [44]. PCR was carried out with EliZyme™ HS FAST MIX Red Master Mix (Elisabeth Pharmacon, Brno, Czech Republic) under the conditions described by Mahayri et al. (2024) [45]. The resulting amplicons were assessed for size and purity by 1.5% agarose gel electrophoresis and subsequently purified with the Monarch^®^ PCR & DNA Cleanup Kit (New England BioLabs, Ipswich, MA, USA).

### 2.4. Library Preparation and Next-Generation Sequencing

High-throughput sequencing was done as described previously [46]. Briefly, to prepare the libraries, NEBNext Fast DNA Library Prep Set for Ion Torrent (New England BioLabs, Ipswich, MA, USA) was used alongside the Ion Xpress Barcode Adapters 1–96 Kit (Thermo Fisher Scientific, Waltham, MA, USA). The amplicons’ size was assessed using the 2100 Bioanalyzer Instrument (Agilent Technologies, Santa Clara, CA, USA). Equimolar pooling of libraries was performed based on the concentrations measured with the KAPA Library Quantification Kit (KAPA Biosystems, Roche, Pleasanton, CA, USA). Emulsion PCR and template enrichment were carried out on the OneTouch™ 2 instrument using the Ion PGM™ HiQ™ View OT2 Kit-400 (Thermo Fisher Scientific, Waltham, MA, USA). Finally, sequencing was performed on the Ion Torrent PGM™ System (Thermo Fisher Scientific, Waltham, MA, USA) using the Ion PGM™ Hi-Q™ View Sequencing solutions kit and the Ion 316™ Chip v2 BC according to the manufacturer’s protocols.

### 2.5. Bioinformatic and Statistical Analysis

Raw sequencing reads obtained from the Ion Torrent platform underwent initial quality filtering to eliminate low-quality and polyclonal sequences. The sequences were obtained in FASTQ format and analyzed using QIIME2 software (version 2020.2) [47]. Quality filtering, trimming, and denoising were applied to the sequences using DADA2, and chimeras were removed [48]. The dataset was normalized through rarefaction, subsampling each sample to a minimum uniform depth of 5998 reads, corresponding to the lowest number of quality-filtered reads obtained among all samples. Rarefaction curves confirmed adequate coverage, as they reached a plateau, indicating that most of the microbial diversity was captured (Figure S1). The number of raw reads and filtered high-quality reads for each sample is reported in Table S3. Sequences were subsequently clustered into Amplicon Sequence Variants (ASVs) via VSEARCH. Taxonomic assignment was then executed through a BLAST search against version 138 of the SILVA database using a 99% similarity threshold [49]. Non-bacterial sequences were removed during sequence processing. The difference in Firmicutes/Bacteroidota (F/B) ratio between groups was assessed using the Kruskal–Wallis test. The bacterial diversity was assessed using alpha diversity indices such as Chao1, Faith’s Phylogenetic Diversity and Shannon Entropy. The comparison between groups was done using the non-parametric Kruskal–Wallis H test and visualized using qiime2R, tidyverse, ggplot2, ggpubr and RColorBrewer packages in RStudio (version 4.4.3). Beta diversity was determined using the unweighted UniFrac distance matrix. Community clustering was visualized through principal coordinate analysis (PCoA), with plots generated in RStudio version 4.5.3 using the qiime2R, ape, ggplot2, and ggpubr packages. To evaluate statistical variations between groups, a permutational multivariate analysis of variance (PERMANOVA) was executed with 999 permutations. Group dispersion homogeneity was subsequently assessed using the PERMDISP test. Additionally, the dplyr package in RStudio and a Venn Diagram were utilized to analyze and illustrate the quantity of unique and shared bacterial genera across groups. Prominent phylotypes within the differentially abundant taxa were identified via the Linear Discriminant Analysis Effect Size (LEfSe) algorithm [50] on the MicrobiomeAnalyst 2.0 platform [51]. Significant differences in taxonomic abundance between groups were detected using the Kruskal–Wallis rank sum test. To control false positives, the false discovery rate (FDR) correction was applied. The LEfSe analysis was performed with the following parameters: *p* < 0.05; FDR < 0.05 and a minimum LDA score = 2.0. The differentially abundant taxa were plotted using ggplot2 and dplyr packages in RStudio. To assess the functional potential of the microbial communities, functional profiling was performed using the Tax4Fun tool [52], which was specifically developed for SILVA database via the MicrobiomeAnalyst 2.0 platform [51]. Default data filtering settings were applied prior to statistical analysis: a low-count filter (minimum count of 4, with the feature required to reach this count in at least 20% of samples) removed rare, low-abundance KOs, and a low-variance filter based on the interquartile range removed uninformative, low-variance features. Differentially abundant KEGG Orthologous groups (KOs) between the two dietary groups were identified using the Kruskal–Wallis test. False discovery rate (FDR) correction was applied using the Benjamini–Hochberg method, and KOs with an adjusted *p*-value (*p* < 0.05) were considered statistically significant. The result was visualized using a volcano plot, created with the ggplot2, dplyr and ggrastr packages in RStudio. The functional genes were categorized into KEGG pathways at the different subclass levels 1, 2, and 3. The Kruskal–Wallis test was then used to determine significant differences between the groups for each pathway (*p* < 0.05). Based on these results, a bar chart illustrating the significant pathways at Level 3 was created using the tidyverse, ggpubr, ggplot2, and ggrastr packages in RStudio. The sequence information was deposited in the Sequence Read Archive under accession number PRJNA1250844.

## 3. Results

### 3.1. Alpha and Beta Diversity

**Figure 1:**
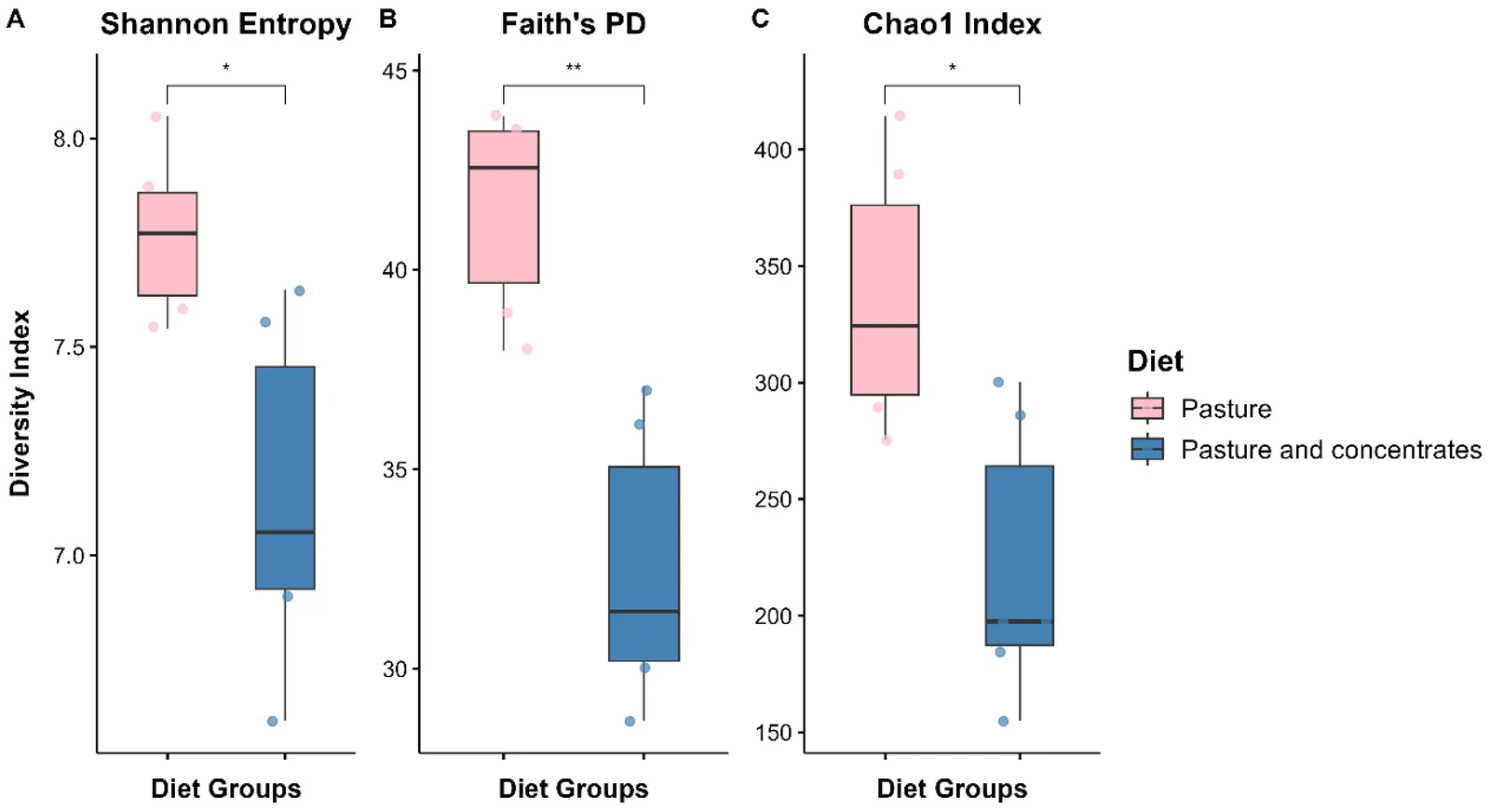
Comparison of diversity indices for the fecal bacterial community of two groups of goats fed different diets. (**A**) Bacterial richness and evenness estimated by Shannon entropy, (**B**) bacterial diversity estimated by Faith’s phylogenetic distance, (**C**) bacterial richness estimated by the Chao1 index. * indicates a significant difference (*p* < 0.05), ** indicates a highly significant difference (*p* < 0.01).

**Figure 2:**
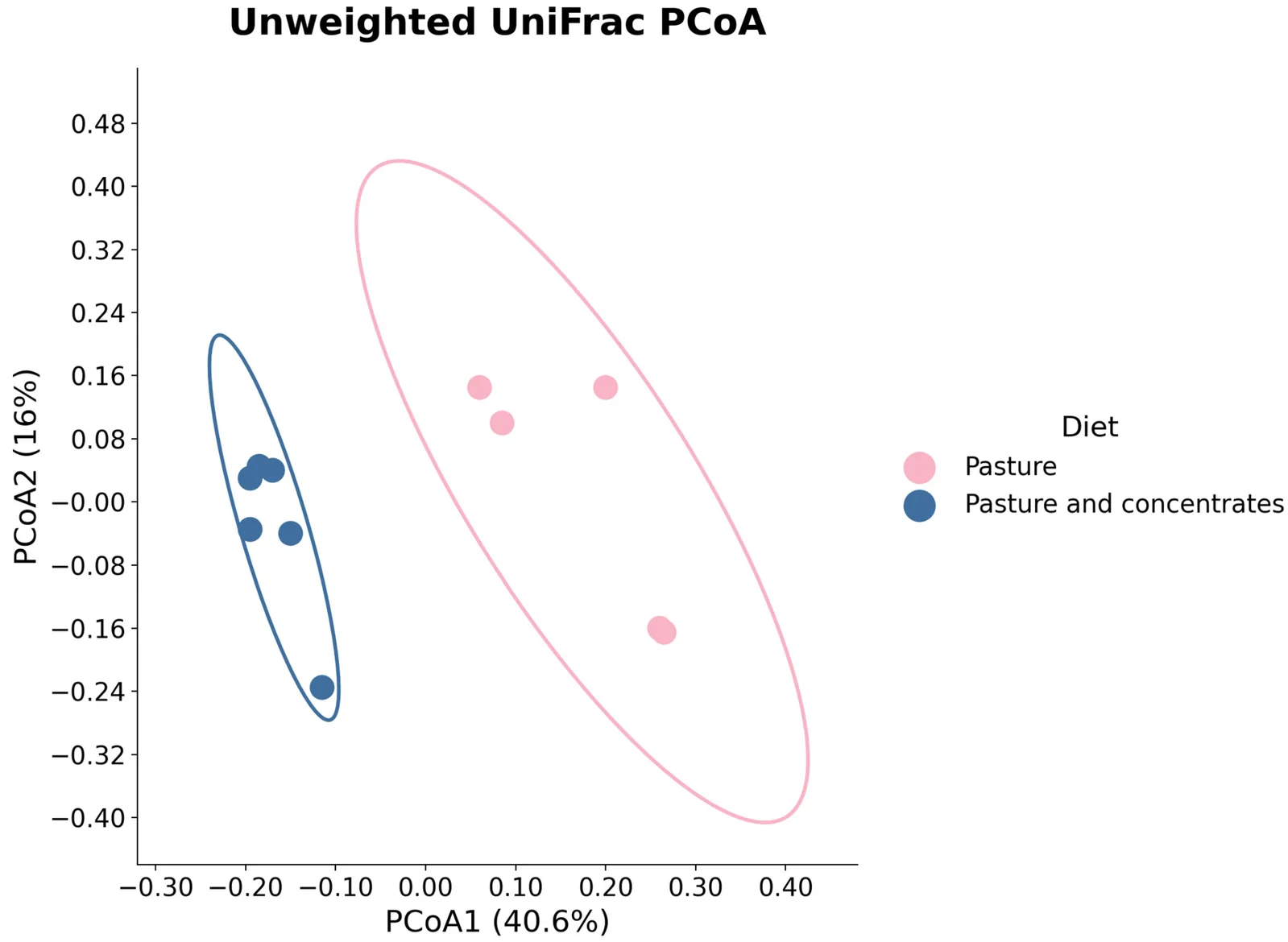
Principal Coordinate Analysis (PCoA) showing the unweighted UniFrac distance matrix for fecal bacterial communities between the pasture-fed (pink) and pasture with concentrates-fed (blue) goat groups. Individual samples are represented by distinct dots, with the percentage of variance displayed on each axis. Statistical significance between groups is denoted by PERMANOVA *p* = 0.005.

Both qualitative and quantitative evaluations of the fecal bacterial population structure in goats on varying diets were conducted to assess species richness, evenness, and phylogenetic diversity. Statistically significant variations regarding Faith’s phylogenetic distance, the Chao1 index, and Shannon entropy were detected between the GC and GP groups. This analysis demonstrated a reduced diversity level within the samples collected from grazing goats supplemented with concentrate (Figure 1). A summary of these alpha diversity findings is provided in Table S4. For beta diversity evaluation, variations among the bacterial communities were established utilizing an unweighted UniFrac distance matrix. A Principal Coordinate Analysis (PCoA) plot (Figure 2) shows spatial separation of the samples. Statistical analysis revealed a significant difference between the groups (PERMANOVA *p* = 0.005). The PERMDISP test confirmed homogeneity of dispersion across groups (*p* = 0.196).

**Figure 3:**
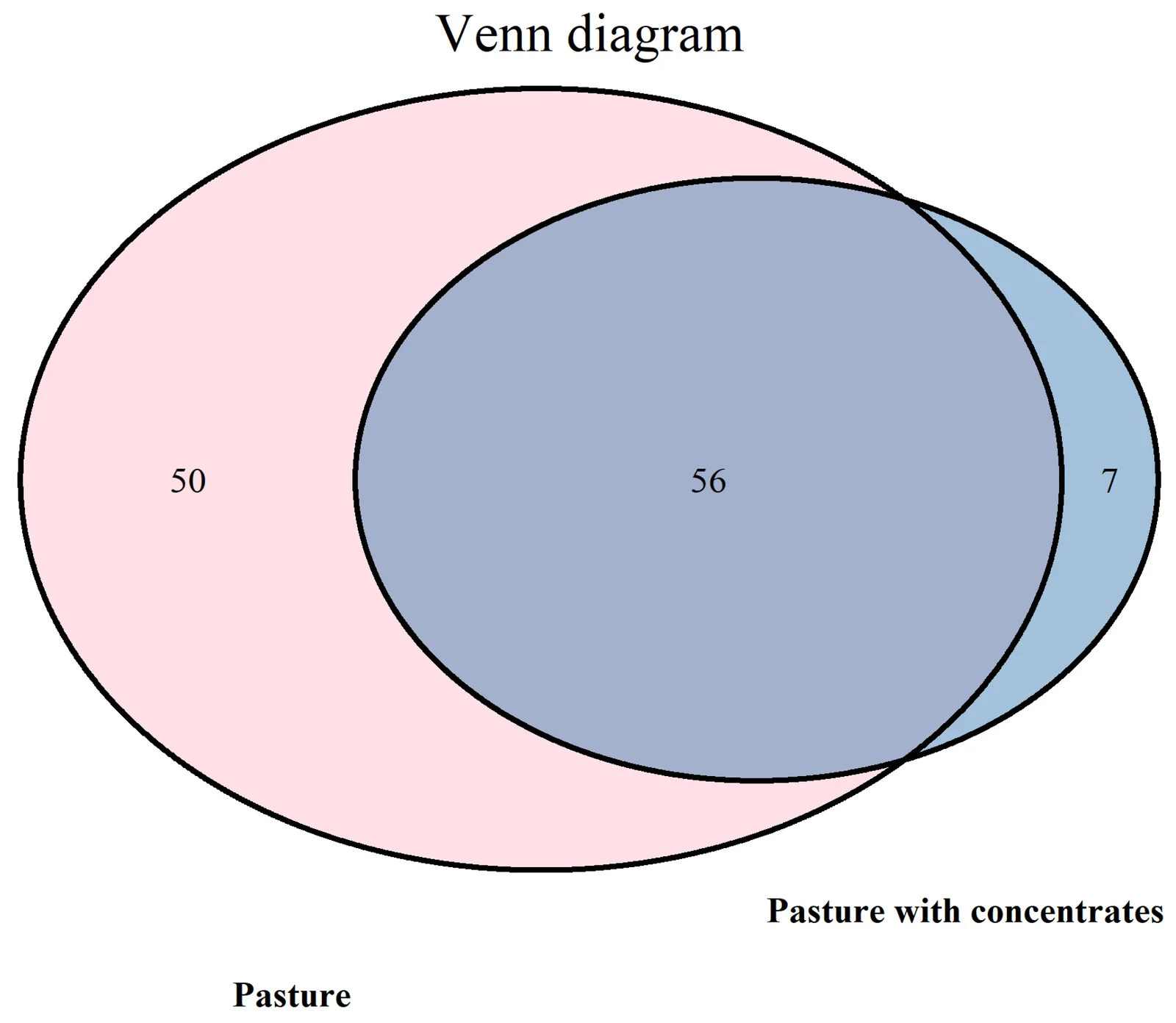
Venn diagram representation of the bacterial genera shared between the fecal samples from two groups of goats showing the unique and shared (overlapping region) genera in the samples of goats on pasture (pink color) and grazing goats fed concentrates (blue color).

Venn diagram analysis of shared and unique taxa is presented at the genus level (Figure 3), the resolution at which the presence and absence differences between dietary groups are most biologically informative. The results showed notable differences in microbial richness between the two groups. Fecal samples of goats shared fifty-six genera; the GP group contained fifty unique genera, while the GC group contained only seven unique genera. These results demonstrate the significant effect of diet on fecal bacterial diversity and indicate that the pasture-based diet supports a broader range of microbial species and contributes to greater microbial diversity.

### 3.2. Taxonomic Composition

In total, the bacterial community in the feces of goats consisted of 12 phyla (including 149 bacterial phylotypes). The fecal composition was dominated by bacteria belonging to Firmicutes (GP: 74.87 ± 3.58%; GC: 82.57 ± 1.88%), followed by Bacteroidota (GP: 22.74 ± 3.70%; GC: 16.44 ± 1.52%). Firmicutes-to-Bacteroidota ratio (F/B) showed a significant difference between the dietary groups, with a higher ratio observed in the concentrate-supplemented group GC (F/B ratio = 5) compared to the grazing group GP (F/B ratio = 3.3) (*p* = 0.037; Figure S2). The abundances of the phyla Desulfobacterota, Verrucomicrobiota, Planctomycetota, Spirochaetota, Proteobacteria, Cyanobacteria, Actinobacteriota, Campilobacterota, Patescibacteria, and Fibrobacterota were low (<0.6%).

**Figure 4:**
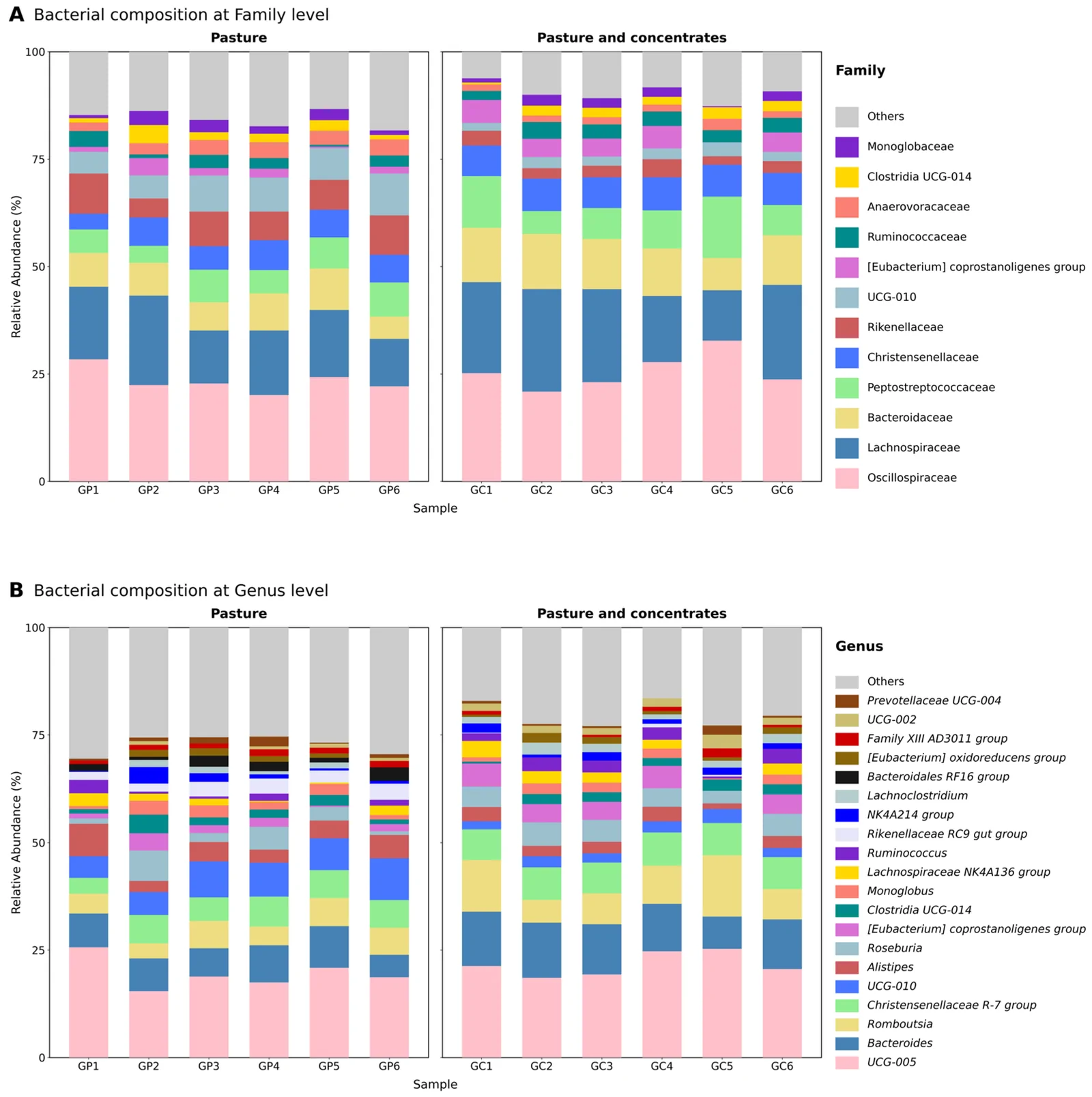
Relative abundance of the fecal bacteria in the two groups of goats fed different diets at (**A**) family level (top 12 families) and (**B**) genus level (top 20 genera). The taxa with lower relative abundances (<1%) are grouped as “Others”.

Regardless of diet, the major order of Firmicutes was Oscillospirales, mainly represented at the family level by Oscillospiraceae (GP: 23.39 ± 2.84%; GC: 25.60 ± 4.17%), followed by Lachnospirales, which was represented at the family level by Lachnospiraceae (GP: 15.29 ± 3.47%; GC: 19.31 ± 4.72%). The main order of Bacteroidota was Bacteroidales, represented at the family level by Bacteroidaceae (GP: 7.60 ± 1.56%; GC: 11.22 ± 1.95%), as shown in Figure 3. At the genus level, the most abundant genera were *UCG-005* (Oscillospiraceae family), *Bacteroides*, *Romboutsia*, *Christensenellaceae* R-7 group, *UCG-010* (Oscillospirales; UCG-010 family), *Alistipes*, and *Roseburia*, which together accounted for 53.46% of the sequences in goats fed pasture and 59.07% in goats fed pasture with concentrates. The 12 most abundant bacterial families and the 20 most abundant bacterial genera are shown in Figure 4. Bacterial taxa with lower relative abundances (<1%) are summarized as “Others”. The relative abundance of the bacterial taxa across various taxonomic ranks is listed in Table S5. 

### 3.3. Determination of Taxonomic Biomarkers

**Figure 5:**
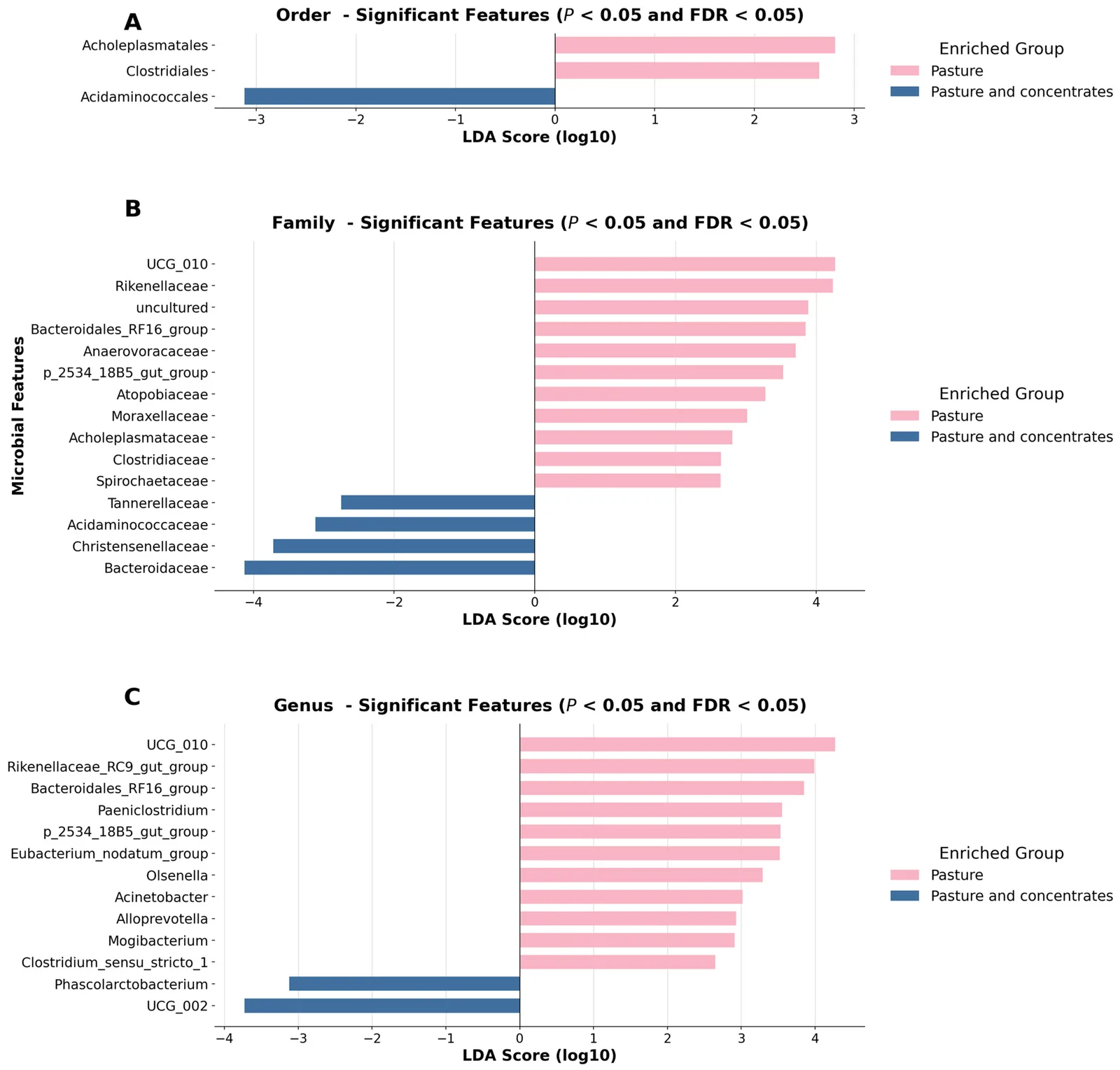
Linear discriminant analysis (LDA) scores for 3 bacterial orders (**A**), 15 bacterial families (**B**), and 13 bacterial genera (**C**), with significantly different abundances in the grazing group (GP) and the group supplemented with concentrates (GC). Logarithmic (log10) LDA scores are represented by bar length, with specific thresholds indicated by vertical dotted lines. Enriched bacterial taxa within the GC group are denoted by negative values (blue bars), whereas positive values (pink bars) signify taxa that are overabundant in the GP group.

Linear Discriminant Analysis Effect Size (LEfSe) identified 31 taxonomic biomarkers (Figure 5 and Table S6), with significant abundance differences between the two goat groups, with 24 taxa higher in the grazing group (GP) and 7 in the supplementation group (GC). In the GP group, the increased order Clostridiales (LDA score > 2.5) comprised the significantly more abundant family Clostridiaceae and the genus *Clostridium sensu stricto 1*. The increased order Acholeplasmatales (LDA score > 2.5) included the increased family Acholeplasmataceae. The increased families UCG-010 (order Oscillospirales), Rikenellaceae, Bacteroidales RF16 group, Anaerovoracaceae, p-2534-18B5 gut group (order Bacteroidales), Atopobiaceae and Moraxellaceae included the more abundant genera *UCG-010*, *Rikenellaceae* RC9 gut group, *Bacteroidales* RF16 group, *Eubacterium* nodatum group, *p-2534-18B5* gut group, *Olsenella* and *Acinetobacter*. The family Spirochaetaceae and the genera *Paeniclostridium* (family Peptostreptococcaceae), *Alloprevotella* (family Prevotellaceae) and *Mogibacterium* (family Anaerovoracaceae) had significantly higher abundances in the fecal content of the goats fed pasture and concentrates. In the GC group, the increased order Acidaminococcales (LDA > 3.0) included the increased family Acidaminococcaceae and the increased genus *Phascolarctobacterium*. The families Tannerellaceae, Christensenellaceae, Bacteroidaceae, and the genus *UCG-002* (family Succinivibrionaceae) were also significantly more abundant.

### 3.4. Functional Pathways Analysis

**Figure 6:**
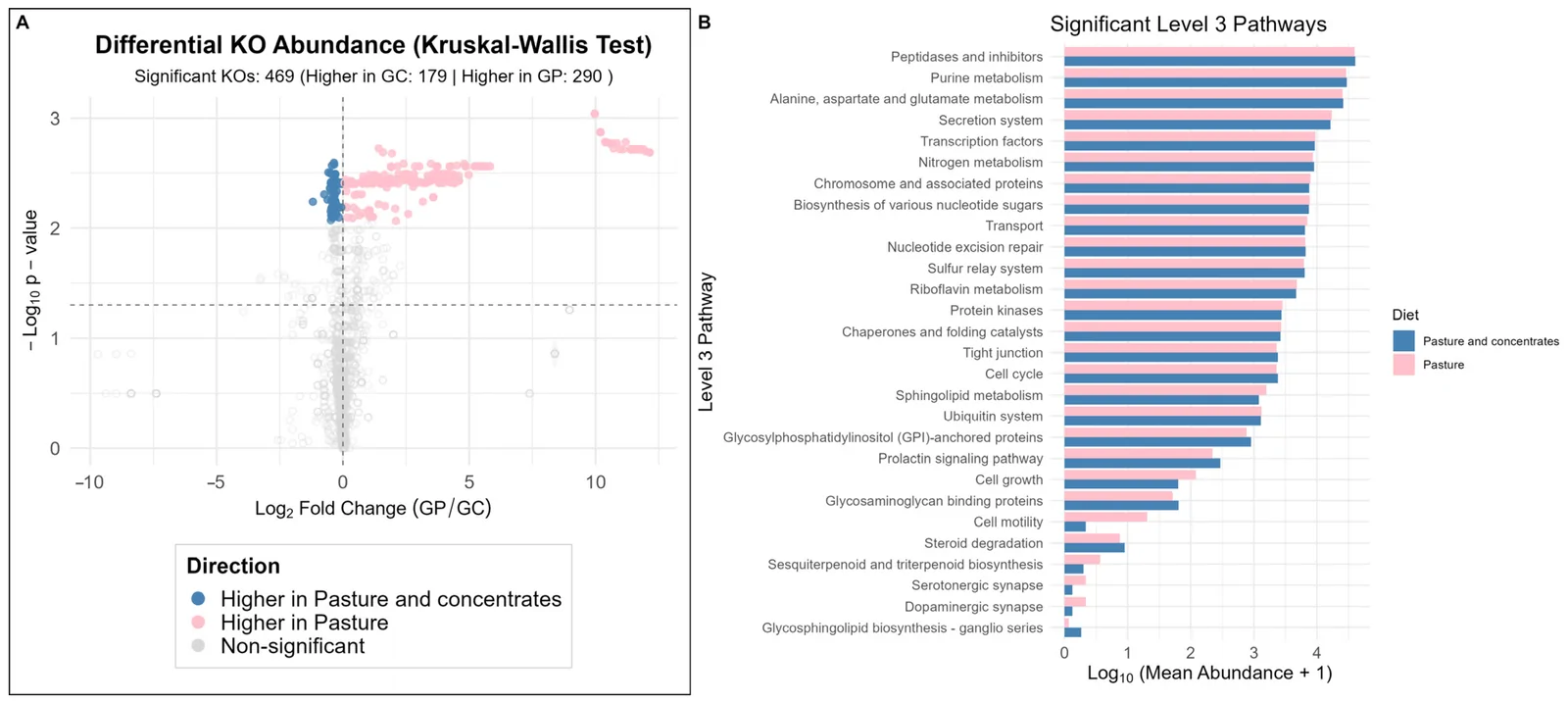
Differential abundance of KEGG Orthology (KO) genes and functions in grazing goats (GP) compared to goats fed a diet supplemented with concentrates (GC). (**A**) Volcano plot illustrating significantly different KEGG Orthologs (KOs) between dietary groups based on a Kruskal–Wallis test with FDR correction (adjusted *p* < 0.05). The *x*-axis represents the log_2_ fold change (GP/GC) and the *y*-axis shows the −log_10_ *p*-values. Each dot represents a KO: pink color indicates higher abundance in grazing goats (GP), blue color indicates higher abundance in goats fed corn and pea supplements (GC). Gray color indicates non-significant KOs. (**B**) Bar plot representing the significant KEGG Level 3 functions. The bars are colored according to the dietary group (pink = GP, blue = GC).

To predict the potential functional profiles of the fecal microbiome in goats, KEGG Orthologs (KOs) were assigned based on sequence alignment, and the relative abundances of KEGG functions were then explored. A total of 469 significant KOs were identified, of which 290 were higher in the GP group and 179 in the GC group (Figure 6A). A total of 36 functions were predicted at level 2, while 249 functions were predicted at level 3. Twenty-eight functions were significantly different, 13 of which were higher in the GC group, while 15 were higher in the GP group (Figure 6B). The enriched functions in grazing goats (GP) included cell growth, cell motility, chaperones and folding catalysts, chromosome and associated proteins, dopaminergic synapse, biosynthesis of various nucleotide sugars, protein kinases, riboflavin metabolism, secretion system, serotonergic synapse, sesquiterpenoid and triterpenoid biosynthesis, sphingolipid metabolism, transcription factors, transport, and the ubiquitin system. The pathways enriched in goats supplemented with concentrates covered alanine, aspartate and glutamate metabolism, cell cycle, glycosaminoglycan binding proteins, glycosphingolipid biosynthesis—ganglio series, glycosylphosphatidylinositol (GPI)-anchored proteins, nitrogen metabolism, nucleotide excision repair, peptidases and inhibitors, prolactin signaling pathway, purine metabolism, steroid degradation, sulfur relay system and tight junction. Level 3 KEGG functions with statistically significant differences in abundance between the studied groups of goats are summarized in Table S7.

## 4. Discussion

This study aims to investigate the effects of dietary supplementation with corn and peas (both 100 g/day) on bacterial diversity and structure in the feces of Sarda goats.

Our results show that the significant impact of dietary grain inclusion on the microbiome of the digestive tract is also reflected in fecal samples. The diet supplemented with corn and peas led to a reduction in bacterial diversity. The decrease in bacterial richness, evenness, and phylogenetic diversity may indicate a shift in microbial community structure, which should be interpreted with caution as evidence of impaired gut health, particularly since no physiological or health-related parameters were assessed in this study, which is in agreement with a number of ruminant studies [28,31,33]. On the other hand, greater species richness and bacterial diversity are associated with improved animal health and well-being [53]. Beta diversity also revealed significant differences between the two dietary groups of Sarda goats. This is consistent with the findings of [28,33], who observed a distinct clustering of fecal bacterial profiles of cows fed diets with different forage-to-grain ratios, using the unweighted Unifrac distance matrix, similar to our study. This reduction may reflect the increased availability of readily fermentable carbohydrates provided by corn supplementation. Because dietary composition is one of the strongest drivers of microbial communities in ruminants, even moderate concentrate supplementation may alter competitive interactions among bacterial taxa. By supplying more starch-derived fermentable material, corn can selectively favor fast-growing saccharolytic bacteria while reducing the competitive advantage of taxa specialized in fiber degradation [43,54,55].

The taxonomic composition of the bacterial communities in the fecal samples analyzed in this study was predominantly composed of Firmicutes, followed by Bacteroidota, a pattern widely reported in ruminants [31,56,57,58]. However, the Firmicutes-to-Bacteroidota (F/B) ratio was significantly higher in the GC group, which is supported by previous studies [13,15,59]. Nonetheless, the biological significance of the F/B ratio remains debated and context-dependent. Firmicutes include taxa specialized in fermenting readily available carbohydrates and producing short-chain fatty acids (SCFAs). An increase in their relative abundance therefore indicates a shift toward more efficient energy harvesting from the starch-rich supplemental diet [60,61,62]. At the family level, Oscillospiraceae, Lachnospiraceae, and Bacteroidaceae were dominant, which is consistent with previous studies [63,64,65]. Oscillospiraceae and Lachnospiraceae include butyrate-producing bacteria with anti-inflammatory properties, and they have been shown to increase production of short-chain fatty acids (SCFAs) [66,67]. Members of these families have been shown to contribute to a higher ruminal fermentation efficiency and an improved barrier function of the rumen epithelium, which is crucial for improved growth performance. Strains from the Bacteroidaceae family are assumed to be involved in almost all bacterial functional gene categories in the gut microbiome [68]. Even though they do not directly produce butyrate, studies in humans have shown that they can contribute to the butyrate pool by producing acetate, which can serve as a co-substrate in the butyryl-CoA transferase pathway within gut bacteria [69]. At the genus level, the core fecal microbiome identified is consistent with previous reports [45,70,71,72]. Several taxa were found at higher frequencies in the grazing group of goats (GP), such as *Rikenellaceae RC9* gut group, *Bacteroidales RF16* group, *p-2534-18B5* gut group, *Alloprevotella* and *Mogibacterium*. These genera are generally associated with fiber fermentation and a forage-based diet. Members of the *Rikenellaceae RC9* gut group seem to be strongly linked with the fecal microbiota [72] and are supposed to be important for the digestion of crude fiber in ruminants [73,74]. Decreased content of neutral detergent fiber in the diet of cows resulted in a significant reduction in their relative abundance [54]. Similarly, the *Bacteroidales RF16* group was found to be related to forage consumption and was previously reported as more abundant in Yaks fed high-fiber diets compared to animals fed concentrate [75]. The *p-2534-18B5* gut group may play a role in protein degradation and has been associated with reduced fat deposition in sheep, suggesting a possible role in regulating energy balance [76].

*Alloprevotella* is particularly well adapted to tannin-rich diets and other plant secondary metabolites. Its presence has been reported in animals supplemented with condensed tannins and herbal mixtures, reflecting its adaptability to complex plant-based diets [60]. Increased abundance of *Mogibacterium* in the non-supplemented grazing group of goats in this study does not confirm findings of the study on goats [77] and sheep [78], in which this genus was enhanced in animals fed high-concentrate diets. *Mogibacterium* is known to be non-fermentative and not nitrate-reducing [77], but its increased abundance was found to be associated with high methane yield cattle [79,80]; thus, the role of *Mogibacterium* in the digestive tract of ruminants requires further investigation. The group of goats additionally fed corn and peas (GC) showed higher levels of only two genera, *Phascolarctobacterium* and *UCG-002* (family Succinivibrionaceae). *Phascolarctobacterium* is a propionate and acetate producer that utilizes succinate. Recently, this genus was found to regulate systemic and hindgut antioxidant defenses in dairy cattle [81], indicating a positive effect of this genus. The *UCG-002* belongs to the bacterial family Succinivibrionaceae, which is known to produce succinate and play a role in amino acid metabolism [82]. In some studies, a correlation between increased abundance of *Succinivibrionaceae UCG-002* and low methane emission in ruminants was observed [79,83]. However, the study of [84] indicates that this relationship is not so simple and needs more detailed analysis. It should be noted that several of these functional interpretations derive from studies performed in other animal species or under substantially different dietary conditions; therefore, these associations should be regarded as plausible working hypotheses, rather than demonstrated mechanisms in Sarda goats.

Functional profiling of the microbial communities using KEGG Orthologs (KOs) suggested potential differences in predicted microbial functionality between the two dietary groups. The KEGG pathways analysis at level 3 indicated that grazing goats (GP) showed a higher predicted abundance of pathways associated with cell growth and motility, secretion systems, and transport mechanisms. These findings are consistent with previous studies reporting the suppression of pathways associated with cell motility and signal transduction in goats fed a high-concentrate diet [85]. Predicted enrichment of riboflavin (vitamin B2) metabolic pathways and sphingolipid biosynthesis, both associated with host health, was also observed in goats kept only on pasture. Riboflavin serves as a cofactor in energy metabolism and antioxidant defense systems, such as glutathione peroxidase [86], while sphingolipids play a critical role in modulating cell signaling and maintaining gut mucosal barrier integrity [87]. In addition, molecular chaperones, which are highly conserved proteins facilitating the proper folding and trafficking of other proteins, were more prevalent in pasture-fed goats. These chaperone systems assist in de novo protein folding, complex assembly, and recovery from stress-induced damage [88]. Pathways associated with protein kinases, which are crucial for signal transduction through phosphorylation of target proteins, also appeared enriched in grazing goats, suggesting a potentially increased regulatory capacity in this group [89]. In contrast, goats fed a diet supplemented with concentrates exhibited higher abundances of pathways involved in nitrogen metabolism, alanine/aspartate/glutamate metabolism, purine metabolism, and steroid degradation. This pattern has been shown to reflect microbial adaptation to a protein-rich diet and enhanced ammonia assimilation [90]. Furthermore, signaling pathways associated with tight junctions and the cell cycle were predicted to be more abundant in the concentrate-fed group (GC), which may indicate possible effects of corn and pea supplementation on intestinal epithelial integrity and permeability [91]. It is important to note that these functional profiles are based on 16S rRNA gene–derived KO predictions, reflecting genomic potential, rather than actual gene expression, enzyme activity or metabolic output. This is why the interpretations should be considered indicative, rather than definitive.

One limitation of this study is the relatively small sample size (n = 6 per group). This constraint is primarily due to the challenges of working with live animals, including the complexities of controlled dietary interventions, sample collection, and microbiome sequencing costs. Nevertheless, our analysis revealed statistically significant differences in diversity indices between the studied groups, and the observed effect of size further supports the biological relevance of these findings, demonstrating a clear impact of diet on the fecal microbiome. However, future studies with larger cohorts will be valuable to confirm these patterns and investigate interindividual variation in greater detail. Another limitation is that the chemical composition of the grazed pasture was not determined; however, both groups grazed the same pasture throughout the study, so any variation in forage quality would have affected both groups equally and is unlikely to bias the between-group comparisons. In addition, physiological and production-related parameters were not evaluated alongside microbiome profiling. Therefore, the microbial changes observed should be interpreted exclusively as modifications in fecal microbial ecology, rather than direct evidence of changes in animal performance, health status, or productivity.

## 5. Conclusions

This study demonstrates that dietary supplementation with pea seeds and corn significantly modulates the fecal microbiota composition and predicted functional potential in Sarda goats. The inclusion of concentrates in the diet led to a reduction in microbial alpha diversity and distinct clustering of bacterial communities, as revealed by beta diversity analysis. Taxonomic profiling indicated a shift in the Firmicutes-to-Bacteroidota ratio and differential abundance of key microbial taxa, suggesting that the diet affects both community structure and specific microbial groups. LEfSe analysis identified several bacterial taxa associated with each dietary treatment, and functional prediction suggested possible differences in microbial metabolic potential, including nutrient metabolism and signaling pathways. These findings highlight the impact of diet on the gut microbiota and its suggested functional capabilities, which are limited to fecal microbial ecology. Further investigations integrating microbiome data with physiological and production parameters are needed to determine the broader implications of these microbial changes for animal performance and health.

## Data Availability

The data presented in this study are openly available in the Sequence Read Archive under the accession number PRJNA1250844.

## Supplementary Materials

The following supporting information can be downloaded at: https://www.mdpi.com/article/10.3390/ani16162486/s1, Table S1. Main components of the feed ingredients used in the experiment. Table S2. Main characteristics of the animals, production system and feeding regimen. Table S3. Summary of the number of raw reads and filtered high-quality reads. Table S4. Summary of alpha diversity indices of bacterial community in goats (means ± SD). Table S5. Relative abundance (means ± SD) of bacterial taxa at phylum, class, order, family and genus levels in the feces of goats fed different diets. Table S6. Differentially abundant bacterial taxa identified by linear discriminant analysis effect size (LEfSe) analysis. Table S7. Differential abundance of significant Level 3 KEGG Pathways (means ± SD) between the control group (GP) and the experimental group (GC). Figure S1. Rarefaction curves representing the sequencing depth (number of reads) and the number of ASVs (sequence variants) found in feces of goats fed different diets. Figure S2. Boxplots showing the Firmicutes-to-Bacteroidota ratio (F/B) in the GP and GC groups. This ratio was significantly increased in the GC group (*p* = 0.037) as determined by Kruskal–Wallis test.
