# Supplementary material for: The Dietary Inclusion of Corn and Peas Affects the Fecal Bacterial Diversity and Composition of Sarda Goats
Source: Animals (Basel). 2026 Aug 10;16(16):2486. doi: 10.3390/ani16162486 (PMC13508916; doi:10.3390/ani16162486)
Supplement: Supplementary file 1 [file animals-16-02486-s001.zip › animals-4448898-supplementary.pdf]

Supplementary Material

Table S1. Main components of the feed ingredients used in the experiment.

Feed	Dry Matter	Crude protein %DM	Crude fiber %DM	Ether extract %DM	NDF %DM	ADF %DM	ADL %DM	Total starch %DM	Ash %DM
Hay	86.9	7.5	34,1	1,7	62,7	39,8	5,8	1,1	8,2
Peas	90,01	24,3	6,5	1,4	14,1	6,6	0,5	46,5	3,1
Corn	87,4	8.7	2,5	3,9	12,6	3,0	0,5	74,1	1,3

Table S2. Main characteristics of the animals, production system and feeding regimen.

Group	GP	GC
Number of animals	6	6
Age range	1–3 years	1–3 years
Production system	Semi-extensive	Semi-extensive
Feeding regimen	Pasture + hay	Pasture + hay + corn and peas

Table S3. Summary of the number of raw reads and filtered high-quality reads.

Sample ID	input	filtered	percentage of input passed filter	denoised	non-chimeric	percentage of input non-chimeric
GC1	12836	7731	60.23	7094	6858	53.43
GC2	15929	8715	54.71	8032	7856	49.32
GC3	23168	13185	56.91	12253	11888	51.31
GC4	11002	6704	60.93	6081	5998	54.52
GC5	14553	7393	50.8	6719	6604	45.38
GC6	21981	12405	56.44	11433	11086	50.43
GP1	32207	22901	71.11	21318	20169	62.62
GP2	32439	17240	53.15	15687	15226	46.94
GP3	20758	12897	62.13	11877	11751	56.61
GP4	24923	14796	59.37	13700	13608	54.6
GP5	26220	12716	48.5	11444	11346	43.27
GP6	17172	12034	70.08	10929	10726	62.46

Table S4. Summary of alpha diversity indices of bacterial community in goats (means ± SD).

Index	GP	GC	P value
Shannon Entropy	7.77 ± 0.19	7.14 ± 0.40	0.0163
Faith's PD	41.58 ± 2.53	32.45 ± 3.40	0.00395
Chao1 Index	336.19 ± 55.53	220.09 ± 58.94	0.0163

* Significant difference ($p < 0.05$)

Table S5. Relative abundance (means ± SD) of bacterial taxa at phylum, class, order, family and genus levels in the feces of goats fed different diets.

Taxonomic levels	GP	GC
**Phylum**		
Actinobacteriota	0.54 ± 0.43%	0.20 ± 0.22%
Bacteroidota	22.74 ± 3.70%	16.44 ± 1.52%
Campilobacterota	0.23 ± 0.12%	0.02 ± 0.02%
Cyanobacteria	0.00 ± 0.01%	0.00 ± 0.00%
Desulfobacterota	0.27 ± 0.23%	0.04 ± 0.11%
Fibrobacterota	0.03 ± 0.05%	0.00 ± 0.00%
Firmicutes	74.87 ± 3.58%	82.57 ± 1.88%
Patescibacteria	0.08 ± 0.11%	0.05 ± 0.05%
Planctomycetota	0.50 ± 0.44%	0.50 ± 0.39%
Proteobacteria	0.46 ± 0.31%	0.11 ± 0.09%
Spirochaetota	0.16 ± 0.10%	0.04 ± 0.04%
Verrucomicrobiota	0.12 ± 0.19%	0.04 ± 0.06%
**Class**		
Actinobacteria	0.02 ± 0.04%	0.00 ± 0.00%
Coriobacteriia	0.53 ± 0.44%	0.20 ± 0.22%
Bacteroidia	22.75 ± 3.71%	16.44 ± 1.52%
Campylobacteria	0.23 ± 0.12%	0.02 ± 0.02%
Vampirivibrionia	0.00 ± 0.01%	0.00 ± 0.00%
Desulfovibrionia	0.27 ± 0.23%	0.04 ± 0.11%
Fibrobacteria	0.03 ± 0.05%	0.00 ± 0.00%
Bacilli	2.32 ± 1.04%	1.70 ± 0.36%
Clostridia	72.21 ± 2.87%	80.19 ± 1.69%
Negativicutes	0.32 ± 0.18%	0.68 ± 0.08%
Saccharimonadia	0.09 ± 0.11%	0.05 ± 0.05%
Planctomycetes	0.50 ± 0.44%	0.50 ± 0.39%
Alphaproteobacteria	0.09 ± 0.09%	0.00 ± 0.00%
Gammaproteobacteria	0.38 ± 0.28%	0.11 ± 0.09%
Spirochaetia	0.16 ± 0.10%	0.04 ± 0.04%
Lentisphaeria	0.12 ± 0.19%	0.04 ± 0.06%
**Order**		
Bifidobacteriales	0.02 ± 0.04%	0.00 ± 0.00%
Coriobacteriales	0.53 ± 0.44%	0.20 ± 0.22%
Bacteroidales	22.75 ± 3.71%	16.44 ± 1.52%
Campylobacterales	0.23 ± 0.12%	0.02 ± 0.02%
Gastranaerophilales	0.00 ± 0.01%	0.00 ± 0.00%
Desulfovibrionales	0.27 ± 0.23%	0.04 ± 0.11%
Fibrobacterales	0.03 ± 0.05%	0.00 ± 0.00%
Acholeplasmatales	0.15 ± 0.10%	0.00 ± 0.00%
Bacillales	0.66 ± 1.01%	0.11 ± 0.13%
Erysipelotrichales	0.30 ± 0.21%	0.50 ± 0.42%
Izemoplasmatales	0.35 ± 0.08%	0.14 ± 0.12%
Lactobacillales	0.19 ± 0.23%	0.00 ± 0.00%
RF39	0.67 ± 0.52%	0.94 ± 0.62%
Unclassided order within class Clostridia	0.33 ± 0.19%	0.79 ± 0.66%
Christensenellales	5.94 ± 1.21%	7.41 ± 0.23%
Clostridia_UCG.014	2.09 ± 1.20%	1.98 ± 0.80%
Clostridia_vadinBB60_group	1.23 ± 0.61%	0.34 ± 0.14%

Clostridiales	0.14 ± 0.12%	0.00 ± 0.00%
Lachnospirales	15.29 ± 3.47%	19.31 ± 4.72%
Monoglobales	2.03 ± 1.02%	1.74 ± 0.91%
Oscillospirales	35.50 ± 2.02%	37.13 ± 3.63%
Peptococcales	0.26 ± 0.32%	0.61 ± 0.42%
Peptostreptococcales.Tissierellales	9.41 ± 2.04%	10.89 ± 3.73%
Acidaminococcales	0.32 ± 0.18%	0.68 ± 0.08%
Saccharimonadales	0.09 ± 0.11%	0.05 ± 0.05%
Pirellulales	0.50 ± 0.44%	0.50 ± 0.39%
Rhodospirillales	0.07 ± 0.07%	0.00 ± 0.00%
Rickettsiales	0.02 ± 0.02%	0.00 ± 0.00%
Aeromonadales	0.00 ± 0.00%	0.11 ± 0.09%
Burkholderiales	0.08 ± 0.08%	0.00 ± 0.00%
Pseudomonadales	0.28 ± 0.32%	0.00 ± 0.00%
Xanthomonadales	0.02 ± 0.02%	0.00 ± 0.00%
Spirochaetales	0.16 ± 0.10%	0.04 ± 0.04%
Victivallales	0.12 ± 0.19%	0.04 ± 0.06%
**Family**		
Bifidobacteriaceae	0.02 ± 0.04%	0.00 ± 0.00%
Atopobiaceae	0.53 ± 0.44%	0.00 ± 0.00%
Eggerthellaceae	0.00 ± 0.00%	0.20 ± 0.22%
Bacteroidaceae	7.60 ± 1.56%	11.22 ± 1.95%
Bacteroidales_RF16_group	1.88 ± 0.91%	0.00 ± 0.00%
Barnesiellaceae	0.02 ± 0.04%	0.00 ± 0.00%
F082	0.29 ± 0.23%	0.13 ± 0.14%
M2PB4.65_termite_group	0.06 ± 0.09%	0.00 ± 0.00%
Marinifilaceae	0.01 ± 0.02%	0.00 ± 0.00%
Muribaculaceae	0.12 ± 0.14%	0.14 ± 0.13%
Paludibacteraceae	0.22 ± 0.18%	0.00 ± 0.00%
Prevotellaceae	1.35 ± 0.64%	0.95 ± 1.42%
Rikenellaceae	7.44 ± 1.84%	2.91 ± 0.79%
Tannerellaceae	0.01 ± 0.02%	0.14 ± 0.10%
p.2534.18B5_gut_group	0.98 ± 0.39%	0.00 ± 0.00%
Uncultured family within order Bacteroidales	2.76 ± 1.67%	0.94 ± 0.52%
Campylobacteraceae	0.23 ± 0.12%	0.02 ± 0.02%
Gastranaerophilales	0.00 ± 0.01%	0.00 ± 0.00%
Desulfovibrionaceae	0.27 ± 0.23%	0.04 ± 0.11%
Fibrobacteraceae	0.03 ± 0.05%	0.00 ± 0.00%
Acholeplasmataceae	0.15 ± 0.10%	0.00 ± 0.00%
Bacillaceae	0.02 ± 0.04%	0.11 ± 0.13%
Planococcaceae	0.64 ± 0.99%	0.00 ± 0.00%
Erysipelatoclostridiaceae	0.04 ± 0.04%	0.00 ± 0.00%
Erysipelotrichaceae	0.26 ± 0.18%	0.50 ± 0.42%
Izemoplasmatales	0.35 ± 0.08%	0.14 ± 0.12%
Leuconostocaceae	0.03 ± 0.05%	0.00 ± 0.00%
Streptococcaceae	0.16 ± 0.25%	0.00 ± 0.00%
RF39	0.67 ± 0.52%	0.94 ± 0.62%
Unclassified family within class Clostridia	0.33 ± 0.19%	0.79 ± 0.66%
Christensenellaceae	5.94 ± 1.21%	7.41 ± 0.23%
Clostridia_UCG.014	2.09 ± 1.20%	1.98 ± 0.80%
Clostridia_vadinBB60_group	1.23 ± 0.61%	0.34 ± 0.14%

Clostridiaceae	0.14 ± 0.12%	0.00 ± 0.00%
Lachnospiraceae	15.29 ± 3.47%	19.31 ± 4.72%
Monoglobaceae	2.03 ± 1.02%	1.74 ± 0.91%
Unclassified family within order Oscillospirales	0.37 ± 0.58%	1.54 ± 1.12%
Eubacterium_coprostanoligenes_group	1.81 ± 1.24%	3.92 ± 1.98%
Butyricicoccaceae	0.42 ± 0.47%	0.51 ± 0.44%
Oscillospiraceae	23.39 ± 2.84%	25.60 ± 4.17%
Ruminococcaceae	2.19 ± 1.28%	3.14 ± 0.62%
UCG.010	7.31 ± 1.82%	2.42 ± 0.50%
Peptococcaceae	0.26 ± 0.32%	0.61 ± 0.42%
Anaerovoracaceae	3.13 ± 0.69%	1.76 ± 0.45%
Peptostreptococcaceae	6.27 ± 1.56%	9.13 ± 3.39%
Peptostreptococcales.Tissierellales	0.01 ± 0.03%	0.00 ± 0.00%
Acidaminococcaceae	0.32 ± 0.18%	0.68 ± 0.08%
Saccharimonadaceae	0.09 ± 0.11%	0.05 ± 0.05%
Pirellulaceae	0.50 ± 0.44%	0.50 ± 0.39%
Uncultured family within order Rhodospirillales	0.07 ± 0.07%	0.00 ± 0.00%
Mitochondria	0.02 ± 0.02%	0.00 ± 0.00%
Succinivibrionaceae	0.00 ± 0.00%	0.11 ± 0.09%
Sutterellaceae	0.08 ± 0.08%	0.00 ± 0.00%
Moraxellaceae	0.28 ± 0.32%	0.00 ± 0.00%
Xanthomonadaceae	0.02 ± 0.02%	0.00 ± 0.00%
Spirochaetaceae	0.16 ± 0.10%	0.04 ± 0.04%
Victivallaceae	0.12 ± 0.19%	0.00 ± 0.00%
vadinBE97	0.00 ± 0.00%	0.04 ± 0.06%
**Genus**		
Uncultured genus within family Bifidobacteriaceae	0.02 ± 0.04%	0.00 ± 0.00%
Olsenella	0.53 ± 0.44%	0.00 ± 0.00%
Parvibacter	0.00 ± 0.00%	0.20 ± 0.22%
Bacteroides	7.60 ± 1.56%	11.22 ± 1.95%
Bacteroidales_RF16_group	1.88 ± 0.91%	0.00 ± 0.00%
Uncultured genus within family Barnesiellaceae	0.02 ± 0.04%	0.00 ± 0.00%
F082.g__F082	0.29 ± 0.23%	0.13 ± 0.14%
M2PB4.65_termite_group	0.06 ± 0.09%	0.00 ± 0.00%
Odoribacter	0.01 ± 0.02%	0.00 ± 0.00%
Muribaculaceae	0.12 ± 0.14%	0.14 ± 0.13%
Uncultured genus within family Paludibacteraceae	0.22 ± 0.18%	0.00 ± 0.00%
Alloprevotella	0.25 ± 0.19%	0.00 ± 0.00%
Prevotella	0.00 ± 0.00%	0.28 ± 0.68%
Prevotellaceae_UCG.003	0.09 ± 0.16%	0.00 ± 0.00%
Prevotellaceae_UCG.004	1.01 ± 0.74%	0.67 ± 0.75%
Alistipes	4.51 ± 1.79%	2.64 ± 0.75%
Rikenellaceae_RC9_gut_group	2.88 ± 0.83%	0.22 ± 0.33%
dgA.11_gut_group	0.05 ± 0.08%	0.04 ± 0.11%
Parabacteroides	0.01 ± 0.02%	0.14 ± 0.10%
p.2534.18B5_gut_group	0.98 ± 0.39%	0.00 ± 0.00%
Uncultured genus within order Bacteroidales	2.76 ± 1.67%	0.94 ± 0.52%

Campylobacter	0.23 ± 0.12%	0.02 ± 0.02%
Gastranaerophilales	0.00 ± 0.01%	0.00 ± 0.00%
Mailhella	0.27 ± 0.23%	0.04 ± 0.11%
Fibrobacter	0.03 ± 0.05%	0.00 ± 0.00%
Anaeroplasma	0.08 ± 0.14%	0.00 ± 0.00%
EMP.G18	0.07 ± 0.06%	0.00 ± 0.00%
Bacillus	0.02 ± 0.04%	0.11 ± 0.13%
Lysinibacillus	0.05 ± 0.08%	0.00 ± 0.00%
Solibacillus	0.57 ± 0.93%	0.00 ± 0.00%
Uncultured genus within family Planococcaceae	0.02 ± 0.04%	0.00 ± 0.00%
Uncultured genus within family Erysipelatoclostridiaceae	0.04 ± 0.04%	0.00 ± 0.00%
Turicibacter	0.26 ± 0.18%	0.50 ± 0.42%
Izemoplasmatales	0.35 ± 0.08%	0.14 ± 0.12%
Weissella	0.03 ± 0.05%	0.00 ± 0.00%
Streptococcus	0.16 ± 0.25%	0.00 ± 0.00%
RF39	0.67 ± 0.52%	0.94 ± 0.62%
Unclassified genus within class Clostridia	0.33 ± 0.19%	0.79 ± 0.66%
Christensenellaceae_R.7_group	5.94 ± 1.21%	7.41 ± 0.23%
Clostridia_UCG.014	2.09 ± 1.20%	1.98 ± 0.80%
Clostridia_vadinBB60_group	1.23 ± 0.61%	0.34 ± 0.14%
Clostridium_sensu_stricto_1	0.14 ± 0.12%	0.00 ± 0.00%
Unclassified genus within family Lachnospiraceae	1.77 ± 0.78%	3.30 ± 0.84%
Eubacterium._oxidoreducens_group	0.96 ± 0.78%	1.24 ± 0.65%
Eubacterium._ruminantium_group	0.41 ± 0.35%	0.70 ± 0.35%
Eubacterium._ventriosum_group	0.31 ± 0.59%	0.00 ± 0.00%
Eubacterium._xylanophilum_group	0.03 ± 0.08%	0.00 ± 0.00%
Acetitomaculum	0.33 ± 0.35%	0.00 ± 0.00%
Anaerosporobacter	0.20 ± 0.35%	0.14 ± 0.16%
Blautia	0.67 ± 1.18%	0.00 ± 0.00%
Butyrivibrio	0.10 ± 0.24%	0.00 ± 0.00%
Cellulosilyticum	0.04 ± 0.07%	0.00 ± 0.00%
Coprococcus	0.41 ± 0.59%	0.06 ± 0.14%
Dorea	0.20 ± 0.25%	0.00 ± 0.00%
Lachnoclostridium	0.88 ± 0.75%	1.87 ± 0.58%
Lachnospiraceae_AC2044_group	0.45 ± 0.45%	1.14 ± 0.38%
Lachnospiraceae_FCS020_group	0.24 ± 0.27%	0.00 ± 0.00%
Lachnospiraceae_NC2004_group	0.07 ± 0.13%	0.13 ± 0.15%
Lachnospiraceae_ND3007_group	0.00 ± 0.00%	0.07 ± 0.17%
Lachnospiraceae_NK3A20_group	0.29 ± 0.36%	0.00 ± 0.00%
Lachnospiraceae_NK4A136_group	1.49 ± 1.06%	2.29 ± 1.27%
Lachnospiraceae_UCG.001	0.71 ± 0.32%	0.34 ± 0.27%
Lachnospiraceae_UCG.004	0.07 ± 0.18%	0.00 ± 0.00%
Marvinbryantia	0.21 ± 0.41%	0.00 ± 0.00%
Roseburia	3.28 ± 2.44%	4.59 ± 0.90%
Tyzzerella	0.43 ± 0.77%	0.66 ± 0.59%
Uncultured genus within family Lachnospiraceae	1.74 ± 1.05%	2.78 ± 0.86%
Monoglobus	2.03 ± 1.02%	1.74 ± 0.91%

Unclassified genus within order Oscillospirales	0.37 ± 0.58%	1.54 ± 1.12%
Eubacterium._coprostanoligenes_group	1.81 ± 1.24%	3.92 ± 1.98%
UCG.008	0.08 ± 0.13%	0.00 ± 0.00%
UCG.009	0.35 ± 0.41%	0.51 ± 0.44%
Unclassified genus within family Oscillospiraceae	0.16 ± 0.25%	0.00 ± 0.00%
NK4A214_group	1.37 ± 1.36%	1.47 ± 0.57%
Oscillibacter	0.10 ± 0.24%	0.00 ± 0.00%
UCG.002	0.53 ± 0.42%	1.98 ± 0.62%
UCG.005	19.52 ± 3.52%	21.66 ± 2.77%
Uncultured genus within family Oscillospiraceae	1.69 ± 0.39%	0.51 ± 1.04%
Unclassified genus within family Ruminococcaceae	0.21 ± 0.36%	0.00 ± 0.00%
Candidatus_Soleaferrea	0.23 ± 0.38%	0.36 ± 0.33%
Incertae_Sedis	0.04 ± 0.11%	0.00 ± 0.00%
Ruminococcaceae	0.08 ± 0.18%	0.05 ± 0.12%
Ruminococcus	1.19 ± 1.13%	2.41 ± 1.12%
Uncultured genus within family Ruminococcaceae	0.44 ± 0.63%	0.32 ± 0.67%
UCG.010	7.31 ± 1.82%	2.42 ± 0.50%
Uncultured genus within family Peptococcaceae	0.26 ± 0.32%	0.61 ± 0.42%
Eubacterium._brachy_group	0.64 ± 0.14%	0.68 ± 0.30%
Eubacterium._nodatum_group	0.95 ± 0.28%	0.10 ± 0.12%
Anaerovorax	0.01 ± 0.03%	0.08 ± 0.09%
Family_XIII_AD3011_group	1.23 ± 0.28%	0.83 ± 0.69%
Mogibacterium	0.29 ± 0.14%	0.07 ± 0.08%
Paeniclostridium	0.93 ± 0.48%	0.00 ± 0.00%
Romboutsia	5.30 ± 1.29%	9.13 ± 3.39%
Terrisporobacter	0.04 ± 0.07%	0.00 ± 0.00%
Anaerococcus	0.01 ± 0.02%	0.00 ± 0.00%
Finegoldia	0.00 ± 0.01%	0.00 ± 0.00%
Phascolarctobacterium	0.32 ± 0.18%	0.68 ± 0.08%
Candidatus_Saccharimonas	0.09 ± 0.11%	0.05 ± 0.05%
p.1088.a5_gut_group	0.50 ± 0.44%	0.50 ± 0.39%
Uncultured genus within order Rhodospirillales	0.07 ± 0.07%	0.00 ± 0.00%
Mitochondria	0.02 ± 0.02%	0.00 ± 0.00%
Ruminobacter	0.00 ± 0.00%	0.06 ± 0.06%
Succinivibrionaceae_UCG.002	0.00 ± 0.00%	0.04 ± 0.04%
Parasutterella	0.06 ± 0.06%	0.00 ± 0.00%
Sutterella	0.02 ± 0.04%	0.00 ± 0.00%
Acinetobacter	0.28 ± 0.32%	0.00 ± 0.00%
Stenotrophomonas	0.02 ± 0.02%	0.00 ± 0.00%
Treponema	0.16 ± 0.10%	0.04 ± 0.04%
Victivallis	0.12 ± 0.19%	0.00 ± 0.00%
vadinBE97	0.00 ± 0.00%	0.04 ± 0.06%

Table S6. Differentially abundant bacterial taxa identified by linear discriminant analysis effect size (LEfSe) analysis.

Taxa	Corresponding family or order	P-value	FDR	LDA score	Increase
**Bacterial orders**					
Acholeplasmatales		0.002093	0.033488	2.81	GP
Clostridiales		0.002093	0.033488	2.65	GP
Acidaminococcales		0.003948	0.042109	-3.12	GC
**Bacterial Families**					
Atopobiaceae	Coriobacteriales	0.002093	0.021767	3.28	GP
Acholeplasmataceae	Acholeplasmatales	0.002093	0.021767	2.81	GP
p_2534_18B5_gut_group	Bacteroidales	0.002093	0.021767	3.53	GP
Uncultured	Bacteroidales	0.002096	0.021768	3.89	GP
Clostridiaceae	Clostridiales	0.002093	0.021767	2.65	GP
Bacteroidales_RF16_group	Bacteroidales_RF16_group	0.002093	0.021767	3.85	GP
Acidaminococcaceae	Acidaminococcales	0.003948	0.02566	-3.12	GC
UCG_010	Oscillospirales	0.003948	0.02566	4.27	GP
Rikenellaceae	Bacteroidales	0.003948	0.02566	4.24	GP
Moraxellaceae	Pseudomonadales	0.007397	0.038465	3.02	GP
Spirochaetaceae	Spirochaetales	0.009876	0.038649	2.64	GP
Christensenellaceae	Christensenellales	0.010406	0.038649	-3.72	GC
Anaerovoracaceae	Peptostreptococcales-Tissierellales	0.010406	0.038649	3.71	GP
Bacteroidaceae	Bacteroidales	0.010406	0.038649	-4.13	GC
Tannerellaceae	Bacteroidales	0.013195	0.045743	-2.75	GC
**Bacterial Genera**					
Olsenella	Atopobiaceae	0.002093	0.029279	3.29	GP
Paeniclostridium	Peptostreptococcaceae	0.002093	0.029279	3.55	GP
p_2534_18B5_gut_group	p_2534_18B5_gut_group	0.002093	0.029279	3.53	GP
Alloprevotella	Prevotellaceae	0.002093	0.029279	2.93	GP
Clostridium_sensu_stricto_1	Clostridiaceae	0.002093	0.029279	2.65	GP
Bacteroidales_RF16_group	Bacteroidales_RF16_group	0.002093	0.029279	3.85	GP
Eubacterium_nodatum_group	Anaerovoracaceae	0.0037	0.029279	3.52	GP
Rikenellaceae_RC9_gut_group	Rikenellaceae	0.0037	0.029279	3.99	GP
Mogibacterium	Anaerovoracaceae	0.0037	0.029279	2.91	GP
UCG_002	Succinivibrionaceae	0.003885	0.029279	-3.73	GC
UCG_010	UCG_010	0.003948	0.029279	4.27	GP
Phascolarctobacterium	Acidaminococcaceae	0.003948	0.029279	-3.12	GC
Acinetobacter	Moraxellaceae	0.007397	0.047025	3.02	GP

Table S7. Differential abundance of significant Level 3 KEGG Pathways (means ± SD) between the control group (GP) and the experimental group (GC).

Pathway Name (Level 3)	GC	GP	P-value
Cell motility	1.167 ± 0.983	19.5 ± 8.961	0.00335
Glycosylphosphatidylinositol (GPI)-anchored proteins	906 ± 21.279	764 ± 82.433	0.00388
Cell growth	62.5 ± 4.506	119.5 ± 38.599	0.00395
Nucleotide excision repair	6616.5 ± 26.994	6531.667 ± 35.848	0.00395
Prolactin signaling pathway	294.667 ± 33.031	221.333 ± 26.681	0.00395
Secretion system	16373.833 ± 327.762	17062 ± 168.553	0.00395
Sulfur relay system	6364.333 ± 33.116	6238 ± 71.375	0.00395

Glycosaminoglycan binding proteins	63.167 ± 4.792	50.667 ± 6.186	0.00485
Sesquiterpenoid and triterpenoid biosynthesis	1 ± 0.632	2.667 ± 0.816	0.00605
Chaperones and folding catalysts	2631.5 ± 12.534	2701 ± 58.107	0.00639
Biosynthesis of various nucleotide sugars	7430.333 ± 146.221	7724.667 ± 122.363	0.00649
Protein kinases	2724.667 ± 17.818	2830.333 ± 59.244	0.00649
Chromosome and associated proteins	7535.333 ± 80.166	7918.667 ± 243.954	0.01041
Purine metabolism	29750.5 ± 158.461	28999.833 ± 557.884	0.01041
Sphingolipid metabolism	1208.333 ± 73.238	1578 ± 278.29	0.01041
Cell cycle	2413.5 ± 33.483	2278.333 ± 68.164	0.01631
Peptidases and inhibitors	40519.667 ± 294.109	39566.167 ± 629.54	0.01631
Tight junction	2413.5 ± 33.483	2278.333 ± 68.164	0.01631
Transcription factors	9276 ± 59.346	9463.167 ± 133.569	0.02497
Ubiquitin system	1294.167 ± 18.28	1326.667 ± 23.157	0.02497
Glycosphingolipid biosynthesis - ganglio series	0.833 ± 0.408	0.167 ± 0.408	0.02703
Dopaminergic synapse	0.333 ± 0.816	1.167 ± 0.408	0.03057
Serotonergic synapse	0.333 ± 0.816	1.167 ± 0.408	0.03057
Alanine, aspartate and glutamate metabolism	26007.833 ± 210.352	25437.833 ± 495.52	0.03737
Nitrogen metabolism	8978.667 ± 170.848	8639.833 ± 234.665	0.03737
Riboflavin metabolism	4691.5 ± 57.201	4803.5 ± 98.569	0.03737
Transport	6453 ± 255.713	6998.667 ± 363.96	0.03737
Steroid degradation	8 ± 0.632	6.5 ± 1.378	0.04843

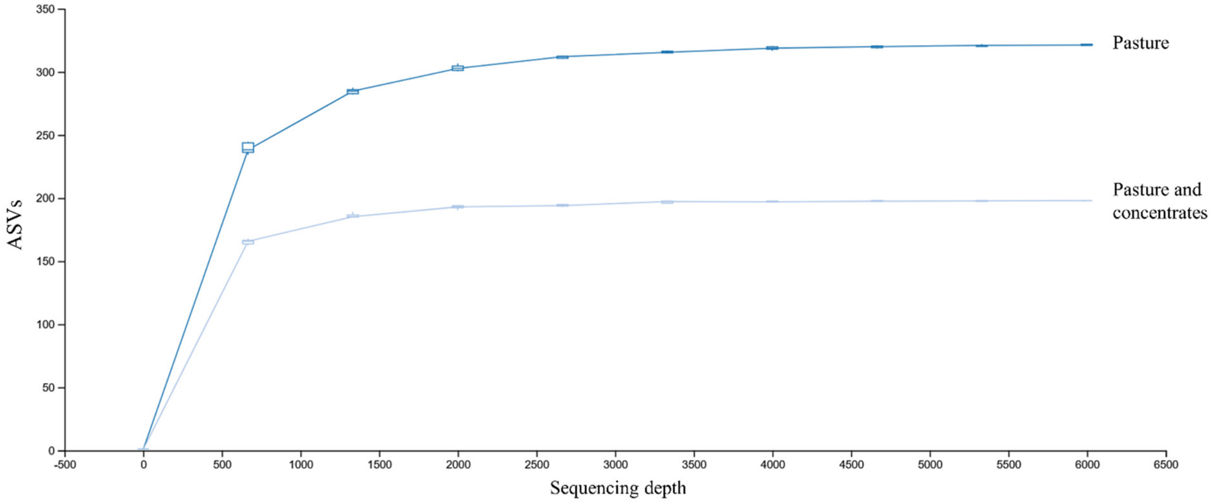

Figure S1. Rarefaction curves representing the sequencing depth (number of reads) and the number of ASVs (sequence variants) found in feces of goats fed different diets.

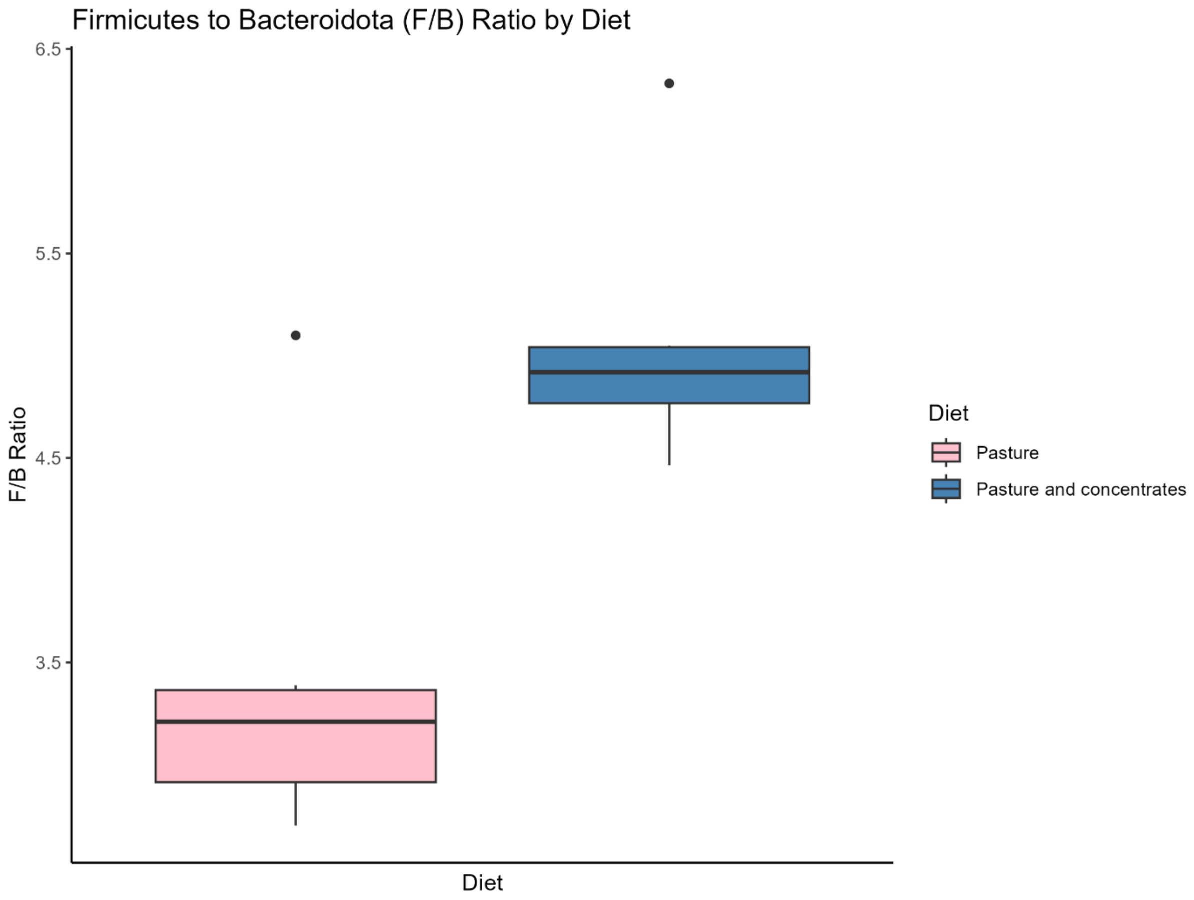

Figure S2. Boxplots showing the Firmicutes-to-Bacteroidota ratio (F/B) in the GP and GC groups. This ratio was significantly increased in the GC group (P = 0.037) as determined by Kruskal-Wallis test.
